# Beneficial effects of inorganic nitrate/nitrite in type 2 diabetes and its complications

**DOI:** 10.1186/s12986-015-0013-6

**Published:** 2015-05-16

**Authors:** Zahra Bahadoran, Asghar Ghasemi, Parvin Mirmiran, Fereidoun Azizi, Farzad Hadaegh

**Affiliations:** Nutrition and Endocrine Research Center, and Obesity Research Center, Research Institute for Endocrine Sciences, Shahid Beheshti University of Medical Sciences, Tehran, Iran; Endocrine Physiology Research Center, Research Institute for Endocrine Sciences, Shahid Beheshti University of Medical Sciences, Tehran, Iran; Endocrine Research Center, Research Institute for Endocrine Sciences, Shahid Beheshti University of Medical Sciences, Tehran, Iran; Department of Clinical Nutrition and Dietetics, Faculty of Nutrition Sciences and Food Technology, National Nutrition and Food Technology Research Institute, Shahid Beheshti University of Medical Sciences, Tehran, Iran; Prevention of Metabolic Disorders Research Center, Research Institute for Endocrine Sciences, Shahid Beheshti University of Medical Sciences, No. 24, Sahid-Erabi St, Yemen St, Chamran Exp, 19395-4763 Tehran, Iran

**Keywords:** Type 2 diabetes, Insulin resistance, Inorganic nitrate and nitrite, Nitric oxide

## Abstract

**Background and aim:**

The ability of inorganic nitrate and nitrite to convert to nitric oxide (NO), and some of its properties e.g. regulation of glucose metabolism, vascular homeostasis, and insulin signaling pathway, have recently raised the hypothesis that inorganic nitrate and nitrite could be potential therapeutic agents in type 2 diabetes. In this review, we reviewed experimental and clinical studies investigating the effect of nitrate/nitrite administration on various aspects of type 2 diabetes.

**Findings:**

Studies showed that an altered metabolism of nitrate/nitrite and impaired NO pathway occurs in diabetes which could contribute to its complications. Some important beneficial properties, including regulation of glucose homeostasis and insulin signaling pathway, improvement of insulin resistance and vascular function, hypotensive, hypolipidemic as well as anti-inflammatory and anti-oxidative effects have been observed following administration of inorganic nitrate/nitrite.

**Conclusion:**

It seems that dietary nitrate/nitrite could be a compensatory fuel for a disrupted nitrate/nitrite/NO pathway and related disorders in diabetes. Although some beneficial properties of nitrate/nitrite have been reported by experimental investigations, long-term clinical studies with various doses of inorganic nitrate/nitrite supplementation, are recommended to confirm these effects.

## Introduction

Inorganic nitrate and nitrite are both naturally occurring as well as food additive compounds in the human diet. Vegetables and other plant based foods are the most common sources of dietary intake of nitrate and contribute up to 85 % total dietary nitrate intake; green leafy vegetables including lettuce and spinach, cabbage, rocket, red beetroot, and radish have higher concentrations of nitrate [1]. Drinking water could also provide considerable amounts of nitrate; main sources of dietary nitrite are processed meat and animal food products [1]. Because of the long-term concerns regarding adverse effects of dietary nitrate and nitrite such as acute toxicity, e.g. methemoglobinemia, production of *N*-nitroso compounds, carcinogenic properties and potential anti-thyroid effect, strict regulations and limitations have been legislated for nitrate/nitrite levels of drinking water and the use of nitrate fertilizer and food additives [2].

Over the past two decades, data on a possible association between high nitrate/nitrite exposure and the risk of childhood type 1 diabetes is controversial; Virtanen *et al*. in 1994 showed that dietary intake of nitrite, but not nitrate, was positively related to the risk of type 1 diabetes in Finnish children [3]. Similar associations have also been reported by others [4–6]; some studies however showed no significant association [7]. In contrast, recent investigations have highlighted the beneficial therapeutic effects of nitrate/nitrite against type 2 diabetes, properties attributed to the potential effects of nitrate/nitrite on their ability to convert to nitric oxide [8, 9]. Current evidence reveals that dietary nitrate/nitrite could restore NO homeostasis and maintain the steady-state NO levels in pathological conditions related to a disrupted NO pathway [10]. Some evidence also indicates that nitrate/nitrite-rich foods such as green leafy vegetables play a protective role against type 2 diabetes and cardiovascular disease [11]. It has also been suggested that high-nitrate content in the diet has a beneficial role in prevention of type 2 diabetes and insulin resistance [12], to the best of our knowledge, so far there is no epidemiological study or clinical investigation to confirm or reject this hypothesis.

In this review we discuss the possible link between type 2 diabetes and nitrate/nitrite metabolism and also the potential effects of these compounds on improvement of glycemic control and prevention of type 2 diabetes complications.

## The link of type 2 diabetes and nitrate/nitrite/NO pathway

### Nitrate/nitrite/NO pathway

Currently, NO is considered as a biologically active hormone, with several functional properties, including regulation of vascular homeostasis and blood pressure, inhibition of platelet activation, regulation of energy and lipid metabolism as well as mitochondrial biogenesis, and modification of various physiological pathways [13, 14]. Impaired NO metabolism, especially reduced NO production and NO bioavailability, has been recognized as a risk factor for development of cardiometabolic disorders, especially vascular dysfunction, cardiovascular disease, chronic kidney disease, endocrine disorders, insulin resistance, metabolic syndrome, and type 2 diabetes [15–18].

Both enzymatic and non-enzymatic pathways have been identified for NO production. Conversion of L-arginine to NO via three isoforms of NO synthase (NOS) is the classical, recognized pathway, recent investigations however show that dietary inorganic nitrate/nitrite could act as a substrate or backup system for non-enzymatic endogenous generation of NO; studies have also revealed that administration of nitrate/nitrite is associated with NO-like bioactivity [19, 20]. The ability of nitrate and nitrite for production of NO, have highlighted the physiological importance and novel aspects of the nitrate/nitrite/NO pathway in health and disease [21].

### Impaired nitrate/nitrite/NO pathway and type 2 diabetes

It has been shown that, NO is implicated as a critical signaling molecule in glucose uptake, and inorganic nitrate and nitrite are highlighted as potential therapeutic agents in insulin resistance and type 2 diabetes [22, 23]. Findings from *in vitro* and animal investigations have raised the hypothesis that the nitrate/nitrite/NO pathway plays a plausible role in glucose homeostasis, insulin secretion, and insulin signaling [24]. A significant association between endothelial NO synthase (eNOS) gene polymorphisms and type 2 diabetes, as well as decreased eNOS expression in skeletal muscle, and reduced NO production and bioavailability in insulin resistance states, clearly underlines the role of NO and its metabolites in the development of hyper-insulinemia, insulin resistance, and type 2 diabetes [25–28].

On the other hand, growing evidence indicate undesirable changes in the metabolism of nitrate/nitrite and NO under diabetic condition. The impaired NO pathway in type 2 diabetes has been linked to consequent metabolic disorders and vascular complications [29].

**Fig. 1:**
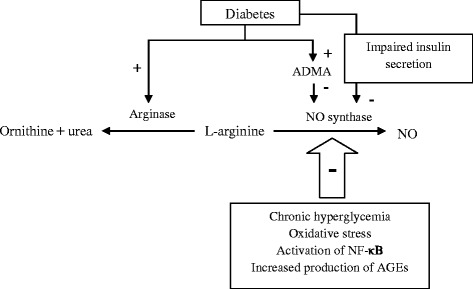
The impaired NO pathway in type 2 diabetes. Several mechanisms have been proposed for impaired NO pathway in diabetic condition 1) Chronic hyperglycemia, increased oxidative stress, NF-κB activation, and accumulation of AGEs 2) Increased plasma levels of ADMA, an endogenous competitive inhibitor of NO synthase 3) Decreased NOS activity and production of NO from L-arginine, increased arginase activity, decreased L-arginine bioavailability and NO synthesis via NOS 4) Impaired insulin secretion and insulin resistance. NO; Nitric oxide, NOS; Nitric oxide synthase, ADMA; Asymmetric dimethylarginine, NF-κB; Nuclear factor κB, AGEs; advanced glycation end products

Some well known mechanisms could describe the impaired NO pathway in type 2 diabetes; first, chronic hyperglycemia, increased oxidative stress and nuclear factor κB (NF-κB) activation, as well as accumulation of advanced glycation end products (AGEs) in diabetic condition are the substantial disruptive factors in the nitrate/nitrite/NO pathway [30]; second, increased plasma levels of asymmetric dimethylarginine (ADMA), an endogenous competitive inhibitor of NO synthase, in diabetic condition is also known as hyperglycemia-induced impairment factor of NO pathway [31]. Third, decreased NOS activity and production of NO from L-arginine as well as increased arginase activity, which leads to decreased L-arginine bioavailability and NO synthesis via NOS, are other disrupted pathways in NO homeostasis in diabetic conditions [32]; finally, since activation of NOS is regulated by insulin and the Akt signaling pathway, impaired insulin secretion and insulin resistance due to diabetes could affect NO synthesis [33] (Fig. 1).

### The altered metabolism of nitrate and nitrite in diabetic condition

There is no consensus regarding the effect of type 2 diabetes on nitrate/nitrite metabolism; both increased and decreased serum and urinary concentration of nitrate/nitrite were observed in type 2 diabetic patients (Table 1). A comparison of 72 type 2 diabetic patients with 60 healthy controls showed a significant lower plasma level of nitrate in diabetic patients (24.9 *vs.* 100 μmol/l) [34]. In a case–control investigation, a lower nitrate/nitrite concentration was observed in type 2 diabetic patients without complications, compared to healthy controls (35 *vs.* 51 μmol/l); but the diabetic group with coronary artery disease showed a higher nitrate/nitrite level (58.9 μmol/l) [35].

**Table 1:** Altered metabolism of nitrate/nitrite in type 2 diabetic patients

Author	Study population	Findings
Francesconi, 2001 [40]	Type 2 diabetic patients	Decreased urinary excretion of nitrate/nitrite after glycemic control; a positive correlation between fasting plasma glucose and urinary nitrate/nitrite
Kawakatso, 2002 [35]	Type 2 diabetic patients with and without complications, compared with healthy controls	Lower nitrate/nitrite concentration in diabetic patients without complications; a higher nitrate/nitrite level in diabetic group with coronary artery disease
Apakkan, 2003 [36]	Type 2 diabetic patients, compared with healthy controls	Higher serum and urine nitrate/nitrite in diabetic patients
Zahedi, 2008 [37]	Subjects with type 2 diabetes and metabolic syndrome, compared with healthy controls	Higher serum nitrate/nitrite concentrations in subjects with type 2 diabetes and metabolic syndrome
Shiekh, 2011 [34]	Type 2 diabetic patients, compared with healthy controls	Lower plasma levels of nitrate in diabetic patients

Increased serum levels of nitrate/nitrite were also found in type 2 diabetic patients; one study on microalbuminuric and normoalbuminuric type 2 diabetic patients, compared to healthy controls, showed a higher serum and urine nitrate/nitrite [36]. In this study, serum nitrate/nitrite concentration was correlated to estimated glomerular filtration rate (eGFR) values and urinary albumin excretion [36]. In another cross-sectional study, serum nitrate/nitrite concentration was significantly higher in subjects with metabolic syndrome (31.9 *vs.* 29.8 μmol/l) and type 2 diabetes (34.6 vs. 30.2 μmol/l) compared to controls [37]. Increased serum levels of nitrate/nitrite and enhanced NO production in diabetes could be a compensatory response to elevated insulin and oxidative stress; inhibition of endothelial NOS (eNOS) and induction of inducible NOS (iNOS) occurred in insulin resistance and inflammatory status, also increased nitrate/nitrite concentrations [38, 39]. A previous research showed that good glycemic control in type 2 diabetic patients decreased urinary nitrate/nitrite fraction and a significant correlation between fasting plasma glucose and the urinary excretion fraction of nitrate/nitrite was observed (*r* = 0.12); this observation could raise the hypothesis that hyperglycemia affects renal nitrate/nitrite handling [40].

### Effects of nitrate/nitrite supplementation on type 2 diabetes and its complications

#### Effects of dietary nitrate on glucose and insulin homeostasis: from experimental to human studies

Findings of animal studies that investigated therapeutic applications of inorganic nitrate or nitrite in diabetic condition, are presented in Table 2 [41–45]. Intraperitoneal injection of sodium nitrite in rats, increased pancreatic islet blood flow without any effect on total blood flow of pancreas, and also enhanced plasma insulin concentration without any effect on glycemia. *In vitro*, sodium nitrite, dose-dependently in a wide concentration range (1–100 μm), stimulated insulin secretion from β cell under basal (5 mM glucose) but not under stimulatory (15 mM) conditions; sodium nitrate had no significant effects on insulin secretion. In this study, a novel indirect insulinotropic action of nitrite was also investigated by induction of the NO/guanylyl cyclase/cGMP signaling pathway at basal glucose conditions [41]. A 4-week administration of 50 mg/l sodium nitrite in diabetic mice also improved fasting glucose levels and reduced insulin concentrations; insulin-independent stimulatory effect of nitrite on glucose transporter type 4 (GLUT4) translocation found in cell-culture examination, suggested that nitrite could improve insulin signaling via restoration of NO-dependent nitrosation of GLUT4 signaling [42]. Long-term administration of nitrate (~100 to 300 g nitrate-rich vegetable, such as spinach, lettuce, or beetroot in humans) in eNOS-deficient mice reversed the prediabetic phenotype; in this study acute doses of inorganic nitrate increased circulating bioactive nitrogen oxides, and chronic administration of nitrate improved glucose homeostasis and decreased pro-insulin/insulin ratios compared to non-treated eNOS-deficient mice [46].

**Table 2:** The effects of nitrate and nitrite on glucose and insulin homeostasis

Author	Animal	Treatment	Outcomes
Carlstrom, 2010 [41]	e-NOS deficient mice	Addition of NaNO_3_ drinking water at a concentration of 85 mg/l for 8–10 weeks	↓ Fasting blood glucose and glycosylated hemoglobin
			↓ Pro-insulin to insulin ratio
			Improved glucose tolerance test
Nystrom, 2012 [42]	Male wistar rats	Intraperitoneal injection of NaNO_2_ or NaNO_3_	↑ Plasma nitrite concentration
			↑ Pancreatic islet blood flow without any effect on total pancreas blood flow
			↑ Plasma insulin concentration without any effect on glycemia
			↑ Insulin secretion from isolated rat islet *in vitro* under basal (5 mM glucose) but not under stimulatory (15 mM glucose) conditions
			Sodium nitrate had no insulinotropic effects at either glucose concentration
Ohtake, 2015 [43]	KKA^y^ diabetic mice	Administration of NO2^−^ in drinking water at a dose of 50 and 150 mg/l for 10 weeks	↓ Glucose level and improve glucose tolerance test at dose of 150 mg/l
			Improved insulin resistance, measured by HOMA-IR at both doses
			↑ GLUT4 translocation in the cell membrane of skeletal muscle
Jiang, 2015 [44]	Db/db diabetic mice	Administration of 50 mg/l NaNO_2_ for 4 weeks	↓ Fasting glucose concentration
			↓ Insulin concentration, ↑ insulin sensitivity and insulin signaling
			↑ GLUT4 translocation in skeletal muscle and adipose tissue *in vitro* at a dose of 10 and 100 μM
Khalifi, 2015 [45]	Streptozotocin-induced diabetic rats	Administration of NaNO_3_ in drinking water at a dose of 100 mg/l for 8 weeks	Restored serum nitrate/nitrite to normal values
			Improved glucose tolerance

NaNo_3_, Sodium nitrate; NaNo_2_, sodium nitrite

**Fig. 2:**
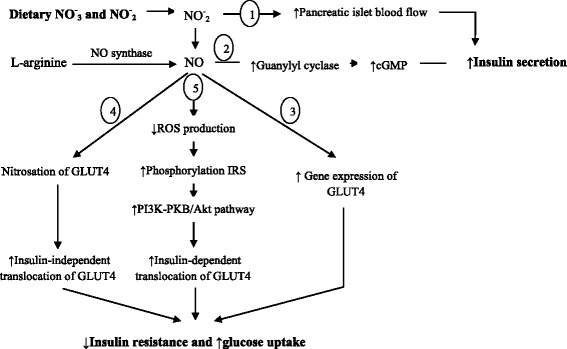
Proposed mechanisms of effects of nitrate/nitrite on glucose homeostasis and insulin signaling. Nitrate/nitrite increase insulin secretion by increasing pancreatic islet blood flow (1) and activation of guanylyl cyclase and cGMP pathway (2). Nitrate/nitrite induce NO production and improve insulin resistance and glucose uptake by increasing gene expression of GLUT4 (3). Nitrate/nitrite increase nitrosation and insulin-independent translocation of GLUT4 and increase glucose uptake by skeletal muscle and adipose tissue (4). Nitrate/nitrite also inhibits production of reactive oxygen species (ROS) in adipoceytes and dephosphorylation activity of protein-tyrosine phosphatase 1B and then facilitate phosphorylation of insulin receptor substrate and subsequently GLUT4 translocation and glucose uptake (5). ROS; Reactive oxygen species, IRS; Insulin receptor substrate, PI3K; Phosphatydal inositol 3 kinase, PKB; Protein kinase B, GLUT4; Glucose transporter 4, NO; Nitric oxide

Figure 2 illustrates the main mechanisms that describe therapeutic effects of nitrate/nitrite on glucose homeostasis and insulin signaling. In brief, this figure shows that nitrate/nitrite could increase insulin secretion by increase pancreatic islet blood flow and activation of guanylyl cyclase and the cGMP pathway; nitrate/nitrite also improve insulin resistance and glucose uptake by increase gene expression of GLUT4. Moreover, nitrate/nitrite increase nitrosation and insulin-independent translocation of GLUT4 and also increase glucose uptake by skeletal muscle and adipose tissue. Nitrate also inhibits the production of reactive oxygen species (ROS) in adipoceytes and dephosphorylation activity of protein-tyrosine phosphatase 1B, thereby facilitating phosphorylation of insulin receptor substrate and subsequently GLUT4 translocation and glucose uptake [43].

Beyond *in vitro* and animal investigations, there is a growing interest for use of clinical trials to determine possible effects of nitrate-rich foods on glycemic control and insulin resistance. However, some beneficial effects were observed following nitrate/nitrite supplementation in humans, but a short intervention-period was an important limitation to confirmation of the expected properties.

Two-week supplementation with high-nitrate beetroot juice (~250 mg nitrate) in type 2 diabetic patients compared to depleted-nitrate beetroot juice (~0.002 mg nitrate), had no significant effect on insulin resistance and whole body glucose disposal, but it did improve simple reaction time as a measure of cognitive function [47, 48]. In this study, median serum concentrations of nitrate (150 *vs.* 31 μmol/, *P* < 0.001) and nitrite (390 *vs.* 232 nmol/l, *P* < 0.001) were significantly higher in the intervention compared to placebo groups [40]. In a randomized cross-over clinical trial of type 2 diabetic patients, acute intra-femoral infusion of sodium nitroprusside (a NO donor compound) compared to verapamil, resulted in greater glucose uptake independent of plasma insulin concentration [46], but infusion of sodium nitroprusside or oral supplementation with nitrate (as isosorbide mononitrate or pentaerythritol tetranitrate) in healthy men had no effect on plasma glucose or insulin levels [49]. Moreover, consumption of beetroot juice had no considerable effect on postprandial plasma glucose and insulin levels in overweight and obese men [50].

#### Effects of nitrate/nitrite on vascular function and blood pressure

It is well known that impaired vascular function, subsequent elevated blood pressure and vascular disease are important undesirable complications of developing insulin resistance and type 2 diabetes; hence therapeutic approach focusing on vascular dysfunction, is a priority in management of type 2 diabetic patients [51, 52].

The potential property of inorganic nitrate and nitrite to convert to NO, a key regulator of vascular homeostasis and a natural vasodilator, has highlighted these anions as therapeutic options in vascular abnormalities and hypertension states [53]. In animal models, administration of sodium nitrate enhanced revascularization, increased migration of bone marrow-derived cells to the ischemic-site, and attenuated apoptosis of regenerative myoblasts in ischemic tissue [54]. Supplementation with sodium nitrate in eNOS-deficient mice with features of metabolic syndrome reduced blood pressure [41]; a similar effect was also observed following treatment with inorganic nitrate in hypertensive and eNOS deficient rat, against hypertension and myocardial ischemia-reperfusion injury [20, 55]; these findings indicate the compensatory effects of exogenous nitrate for a disrupted nitrate/nitrite/NO pathway and related disorders in pathological conditions. Administration of sodium nitrite in high-fat induced hypercholesterolemic rat models had anti-inflammatory effects and inhibits the leukocyte adhesion and emigration and prevented the vascular dysfunction [56].

Findings from clinical studies also suggest that inorganic nitrate could be considered as a supplementary treatment in type 2 diabetes. Dietary supplementation with sodium nitrate at a dose of 150 μmol/kg body weight (~300 g spinach) could improve endothelial dysfunction and vascular stiffness in subjects with moderately increased cardiovascular risk [57]. Other cardiovascular protective effects including anti-platelet activity, hypolipidemic and anti-inflammatory effects, and improved blood flow in hypoxic and ischemic tissue have also been reported following nitrate/nitrite supplementation [53, 58, 59].

Improvement of endothelial dysfunction and a blood pressure lowering effect of nitrate have been observed in several clinical investigations; a recent meta-analysis reported considerable decrease in systolic (−4.4 mm Hg; 95 % CI = −5.9, −2.8; *P* < 0.001) and diastolic blood pressure (−1.1 mm Hg; 95 % CI = −2.2, 0.1; *P* = 0.06) following supplementation with inorganic nitrate and high-nitrate beetroot juice [60]. Findings from another meta-analysis also report the beneficial effects of inorganic nitrate supplementation on vascular function [61], effects which have not yet been confirmed in diabetic patients [47]. In a double-blind, randomized, placebo-controlled crossover trial, 2-weeks supplementation with 250 ml/d beetroot juice (7.5 mmol nitrate/d) or 250 ml/d nitrate-depleted beetroot juice (0.002 mmol nitrate/d), increased plasma concentration of nitrite and nitrate, but no significant changes was observed in systolic and diastolic blood pressures, or in macro- or micro-vascular endothelial function [60].

Data shows that impaired metabolic pathways in diabetes could lead to diminished NO production and bioavailability, inactivated NO and impaired eNOS activity and endothelium-dependent vasodilatation [30, 62], indicating the NO-like activity and blood pressure lowering effects of dietary nitrate supplementation observed in healthy subjects, would be mild in diabetic patients. It should be mentioned here that supplementation with any of the bioactive food components has not yet been confirmed for use in people with diabetes [63].

#### Effects of nitrate/nitrite on energy homeostasis and adipose tissue metabolism

A significant association of serum NO and its metabolites with visceral fat and anthropometric measures, observed in a previous cross-sectional study [64] suggested that nitrate/nitrite/NO pathway may be important in energy homeostasis and adipose tissue metabolism, but the experimental effects of inorganic nitrate/nitrite in this field is still a controversial less documented issue. Although some animal investigations reported reduced weight gain and visceral fat accumulation following nitrate/nitrite administration [41], other studies observed no significant changes in usual food intake or the trend of weight gain at various doses of nitrate and nitrite. Moreover, changes in adipose tissue morphology including decreased adipocyte size were observed after administration of sodium nitrite at doses of 50 and 150 mg/l in drinking water of diabetic rat models [43]. Evidence shows that inorganic nitrate enhances expression of thermogenic genes in both brown and white adipose tissue, increases oxygen consumption and β-oxidation of fatty acids, promotes the browning of white adipose tissue and induces anti-obesity effects [65]. More interestingly, it has been demonstrated that the nitrate/nitrite/NO pathway interacts with mitochondrion biogenesis and metabolism; inorganic nitrate could improve oxidative phosphorylation efficiency (P/O ratio), thermodynamic efficiency, and reduce the expression of ATP/ADP translocase, a protein involved in proton conductance, of the respiratory chain in mitochondria [66].

#### Effects of nitrate/nitrite on lipid metabolism

The favorable effects on lipid and lipoprotein metabolism are other potential properties of inorganic nitrate/nitrite. Administration of sodium nitrate in diabetic rat models significantly decreased serum triglycerides, total cholesterol and LDL-C and increased HDL-C levels [45]. A substantial decrease in TG levels was also observed in e-NOS deficient mice after treatment with inorganic nitrate [35]. In humans, 30-day supplementation with dietary nitrate in subjects at risk of cardiovascular disease normalized TG concentration [29].

#### Other beneficial effects of nitrate/nitrite on diabetes complications

There is growing evidence that nitrate/nitrite supplementation, at a moderate dose (comparable with the normal human diet, provided 100–300 g of nitrate-rich vegetables via daily consumption), has interesting physiological functions which could be beneficial in the prevention of diabetes-related complications. Anti-inflammatory and anti-oxidative effects of nitrate/nitrite as well as preventive properties against micro- and macro-vascular disorders and renal injury are important of these benefits [67]. Treatment with inorganic nitrate could attenuate reduced levels of total antioxidant capacity and catalase activity as well as increase the level of pro-inflammatory cytokines in diabetic condition [45, 67, 68]. Table 3 illustrates the potential beneficial effects of nitrate/nitrite supplementation against renal dysfunction in diabetic and other similar pathological conditions in animal models.

**Table 3:** Beneficial effects of nitrate/nitrite administration on type 2 diabetes complications

Author	Animal	Treatment	Outcomes
Ohtake, 2007 [67]	Streptozotocin-induced diabetic male rats	Administration of nitrite in drinking water for 4 weeks	↓ Serum malondialdehyde concentrations
			↑ Nitrite levels in the kidney
			Improvement in glomerular injury parameters including urinary protein and albumin excretion, histopathological glomerular hypertrophy, and mesangial matrix accumulation
Carlstrom, 2011 [68]	Sprague–Dawley rats subjected to unilateral nephrectomy and chronic high-salt diet	Administration of nitrate at doses of 0.14 and 1.4 g NaNO_3_/kg diet for 10 weeks	↓ Proteinuria
			Improvement of histological renal injury, cardiac hypertrophy and fibrosis
			Restored tissue levels of bioactive nitrogen oxides
			↓ Plasma malondialdehyde concentration
			↓Urinary F2-isoprostanes and 8-hydroxy-2-deoxyguanosine
			Attenuates increased plasma and urinary levels of dimethylarginine (an endogenous NOS inhibitor)
			↑ Serum L-arginine levels

NaNo_3_; Sodium nitrate, NOS; Nitric oxide synthase

### Potential side effects of inorganic nitrate/nitrite

With respect to the reported adverse effects of nitrate/nitrite, including both acute and chronic-subclinical toxicity, and also given the large number of ongoing researches in this field, it is essential to clarify the pharmacokinetics and potential side effects of nitrate/nitrite. Considering the increasing concern regarding high-doses of nitrite supplementation, it appears that the use of nitrate from natural sources is less hazardous [69].

Greenway *et al*. in a randomized crossover study of 12 diabetic patients, assessed the effects of 80 mg sodium nitrite with both immediate release and enteric-coated formulations on methemoglobin, sulfhemoglobin, blood pressure, pulse rate, complete blood count, chemistry panel, reported a good tolerance without any adverse events [70]. In another study, Mohler *et al*. also reported no adverse effect following supplementation with two doses of 40 and 80 mg sodium nitrite in patients with diabetes and peripheral artery disease [71]. However, the potential adverse effects of nitrate to generate peroxynitrite and reactive nitrogen intermediates, has been a main concern for a long time; more recent studies however reported no significant increase in the nitration reaction following supplementation of inorganic nitrate [66].

## Conclusion

Findings from *in vitro* and animal studies indicate that inorganic nitrate/nitrite could be considered as an alternative source of endogenous NO which could enhance the insulin signaling pathway, glucose uptake and attenuate insulin resistance and diabetes complications. Dietary nitrate/nitrite could also attenuate other disrupted NO-dependent pathways including vascular function, regulation of blood pressure, mitochondrial biogenesis and energy homeostasis as well as adipose tissue metabolism, lipid and lipoprotein metabolism, in diabetes. There are many reasons to support the hypothesis whether a nitrate/nitrite rich diets provides an effective nutritional strategy for management of type 2 diabetes and prevention of complications. Long-term interventional studies with various doses and food sources of inorganic nitrate/nitrite in human should be performed to confirm therapeutic properties and detect potential unwanted and adverse effects of these compounds.

## References

[1] Hord NG, Tang Y, Bryan NS (2009). Food sources of nitrates and nitrites: the physiologic context for potential health benefits. Am J Clin Nutr.

[2] Alexander J, Benford D, Cockburn A, Cravedi J, Dogliotti E, Di Domenico A (2008). Opinion of the scientific panel on contaminants in the food chain on a request from the European commission to perform a scientific risk assessment on nitrate in vegetables. EFSA J.

[3] Virtanen SM, Jaakkola L, Rasanen L, Ylonen K, Aro A, Lounamaa R (1994). Nitrate and nitrite intake and the risk for type 1 diabetes in Finnish children. Childhood Diabetes in Finland Study Group. Diabet Med.

[4] Dahlquist GG, Blom LG, Persson LA, Sandstrom AI, Wall SG (1990). Dietary factors and the risk of developing insulin dependent diabetes in childhood. BMJ.

[5] Parslow RC, McKinney PA, Law GR, Staines A, Williams R, Bodansky HJ (1997). Incidence of childhood diabetes mellitus in Yorkshire, northern England, is associated with nitrate in drinking water: an ecological analysis. Diabetologia.

[6] Kostraba JN, Gay EC, Rewers M, Hamman RF (1992). Nitrate levels in community drinking waters and risk of IDDM. An ecological analysis. Diabetes Care.

[7] Winkler C, Mollenhauer U, Hummel S, Bonifacio E, Ziegler AG (2008). Exposure to environmental factors in drinking water: risk of islet autoimmunity and type 1 diabetes–the BABYDIAB study. Horm Metab Res.

[8] Lundberg JO, Carlstrom M, Larsen FJ, Weitzberg E (2011). Roles of dietary inorganic nitrate in cardiovascular health and disease. Cardiovasc Res.

[9] Machha A, Schechter AN (2011). Dietary nitrite and nitrate: a review of potential mechanisms of cardiovascular benefits. Eur J Nutr.

[10] Bryan NS, Fernandez BO, Bauer SM, Garcia-Saura MF, Milsom AB, Rassaf T (2005). Nitrite is a signaling molecule and regulator of gene expression in mammalian tissues. Nat Chem Biol.

[11] Cooper AJ, Forouhi NG, Ye Z, Buijsse B, Arriola L, Balkau B (2012). Fruit and vegetable intake and type 2 diabetes: EPIC-InterAct prospective study and meta-analysis. Eur J Clin Nutr.

[12] Gilchrist M, Benjamin N (2010). Vegetables and diabetes. Is nitrate the answer?. BMJ.

[13] Ghasemi A, Zahediasl S (2011). Is nitric oxide a hormone?. Iran Biomed J.

[14] Knott AB, Bossy-Wetzel E (2010). Impact of nitric oxide on metabolism in health and age-related disease. Diabetes Obes Metab.

[15] Baylis C (2008). Nitric oxide deficiency in chronic kidney disease. Am J Physiol Renal Physiol.

[16] Napoli C, Ignarro LJ (2001). Nitric oxide and atherosclerosis. Nitric Oxide.

[17] Masha A, Dinatale S, Allasia S, Martina V (2011). Role of the decreased nitric oxide bioavailability in the vascular complications of diabetes mellitus. Curr Pharm Biotechnol.

[18] Bahadoran Z, Mirmiran P, Ghasemi A, Kabir A, Azizi F, Hadaegh F (2015). Is dietary nitrate/nitrite exposure a risk factor for development of thyroid abnormality? A systematic review and meta-analysis. Nitric Oxide.

[19] Lundberg JO, Weitzberg E, Gladwin MT (2008). The nitrate-nitrite-nitric oxide pathway in physiology and therapeutics. Nat Rev Drug Discov.

[20] Bryan NS, Calvert JW, Gundewar S, Lefer DJ (2008). Dietary nitrite restores NO homeostasis and is cardioprotective in endothelial nitric oxide synthase-deficient mice. Free Radic Biol Med.

[21] Weitzberg E, Lundberg JO (2013). Novel aspects of dietary nitrate and human health. Annu Rev Nutr.

[22] Ghasemi A, Zahediasl S (2013). Potential therapeutic effects of nitrate/nitrite and type 2 diabetes mellitus. Int J Endocrinol Metab.

[23] Henstridge DC, Drew BG, Formosa MF, Natoli AK, Cameron-Smith D, Duffy SJ (2009). The effect of the nitric oxide donor sodium nitroprusside on glucose uptake in human primary skeletal muscle cells. Nitric Oxide.

[24] Kobayashi J (2015). Nitric oxide and insulin resistance. Mmunoendocrinology.

[25] Monti LD, Barlassina C, Citterio L, Galluccio E, Berzuini C, Setola E (2003). Endothelial nitric oxide synthase polymorphisms are associated with type 2 diabetes and the insulin resistance syndrome. Diabetes.

[26] Angeline T, Krithiga HR, Isabel W, Asirvatham AJ, Poornima A (2011). Endothelial nitric oxide synthase gene polymorphism (G894T) and diabetes mellitus (type II) among South Indians. Oxid Med Cell Longev.

[27] Shimabukuro M, Higa N, Tagawa T, Yamakawa K, Sata M, Ueda S (2013). Defects of vascular nitric oxide bioavailability in subjects with impaired glucose tolerance: a potential link to insulin resistance. Int J Cardiol.

[28] Georgescu A, Popov D, Constantin A, Nemecz M, Alexandru N, Cochior D (2011). Dysfunction of human subcutaneous fat arterioles in obesity alone or obesity associated with Type 2 diabetes. Clin Sci (Lond).

[29] Loscalzo J, Welch G (1995). Nitric oxide and its role in the cardiovascular system. Prog Cardiovasc Dis.

[30] Oelze M, Schuhmacher S, Daiber A (2010). Organic nitrates and nitrate resistance in diabetes: the role of vascular dysfunction and oxidative stress with emphasis on antioxidant properties of pentaerithrityl tetranitrate. Exp Diabetes Res.

[31] Lin KY, Ito A, Asagami T, Tsao PS, Adimoolam S, Kimoto M (2002). Impaired nitric oxide synthase pathway in diabetes mellitus: role of asymmetric dimethylarginine and dimethylarginine dimethylaminohydrolase. Circulation.

[32] Romero DHP MJ, Caldwell RB, Caldwell RW (2006). Does Elevated Arginase Activity Contribute to Diabetes-induced Endothelial Dysfunction?. The Journal of Federation of American Societies for Experimental Biology.

[33] Vincent MA, Montagnani M, Quon MJ (2003). Molecular and physiologic actions of insulin related to production of nitric oxide in vascular endothelium. Curr Diab Rep.

[34] Shiekh GA, Ayub T, Khan SN, Dar R, Andrabi KI (2011). Reduced nitrate level in individuals with hypertension and diabetes. J Cardiovasc Dis Res.

[35] Kawakatso TI M, Kani K, Nakagava A, Hiura M, Hazui H (2002). Plasma Nitrate/Nitrite Concentration in Healthy Population and Patients with Diabetes Mellitus Relationships with Gender, Aging and Diabetic Complications. Bull Osaka Med Coll.

[36] Apakkan Aksun S, Ozmen B, Ozmen D, Parildar Z, Senol B, Habif S (2003). Serum and urinary nitric oxide in Type 2 diabetes with or without microalbuminuria: relation to glomerular hyperfiltration. J Diabetes Complications.

[37] Zahedi Asl S, Ghasemi A, Azizi F (2008). Serum nitric oxide metabolites in subjects with metabolic syndrome. Clin Biochem.

[38] Kagota S, Yamaguchi Y, Tanaka N, Kubota Y, Kobayashi K, Nejime N (2006). Disturbances in nitric oxide/cyclic guanosine monophosphate system in SHR/NDmcr-cp rats, a model of metabolic syndrome. Life Sci.

[39] Scherrer U, Sartori C (2000). Defective nitric oxide synthesis: a link between metabolic insulin resistance, sympathetic overactivity and cardiovascular morbidity. Eur J Endocrinol.

[40] Francesconi F, Mingardi R, de Kreutzenberg S, Avogaro A (2001). Effect of metabolic control on nitrite and nitrate metabolism in type 2 diabetic patients. Clin Exp Pharmacol Physiol.

[41] Carlstrom M, Larsen FJ, Nystrom T, Hezel M, Borniquel S, Weitzberg E (2010). Dietary inorganic nitrate reverses features of metabolic syndrome in endothelial nitric oxide synthase-deficient mice. Proc Natl Acad Sci U S A.

[42] Nystrom T, Ortsater H, Huang Z, Zhang F, Larsen FJ, Weitzberg E (2012). Inorganic nitrite stimulates pancreatic islet blood flow and insulin secretion. Free Radic Biol Med.

[43] Ohtake K, Nakano G, Ehara N, Sonoda K, Ito J, Uchida H (2015). Dietary nitrite supplementation improves insulin resistance in type 2 diabetic KKA(y) mice. Nitric Oxide.

[44] Jiang H, Torregrossa AC, Potts A, Pierini D, Aranke M, Garg HK (2014). Dietary nitrite improves insulin signaling through GLUT4 translocation. Free Radic Biol Med.

[45] Khalifi S, Rahimipour A, Jeddi S, Ghanbari M, Kazerouni F, Ghasemi A (2015). Dietary nitrate improves glucose tolerance and lipid profile in an animal model of hyperglycemia. Nitric Oxide.

[46] Henstridge DC, Kingwell BA, Formosa MF, Drew BG, McConell GK, Duffy SJ (2005). Effects of the nitric oxide donor, sodium nitroprusside, on resting leg glucose uptake in patients with type 2 diabetes. Diabetologia.

[47] Gilchrist M, Winyard PG, Aizawa K, Anning C, Shore A, Benjamin N (2013). Effect of dietary nitrate on blood pressure, endothelial function, and insulin sensitivity in type 2 diabetes. Free Radic Biol Med.

[48] Gilchrist M, Winyard PG, Fulford J, Anning C, Shore AC, Benjamin N (2014). Dietary nitrate supplementation improves reaction time in type 2 diabetes: development and application of a novel nitrate-depleted beetroot juice placebo. Nitric Oxide.

[49] Henstridge DC, Duffy SJ, Formosa MF, Ahimastos AA, Thompson BR, Kingwell BA (2009). Oral nitrate therapy does not affect glucose metabolism in healthy men. Clin Exp Pharmacol Physiol.

[50] Joris PJ, Mensink RP (2013). Beetroot juice improves in overweight and slightly obese men postprandial endothelial function after consumption of a mixed meal. Atherosclerosis.

[51] Schofield I, Malik R, Izzard A, Austin C, Heagerty A (2002). Vascular structural and functional changes in type 2 diabetes mellitus: evidence for the roles of abnormal myogenic responsiveness and dyslipidemia. Circulation.

[52] Creager MA, Luscher TF, Cosentino F, Beckman JA (2003). Diabetes and vascular disease: pathophysiology, clinical consequences, and medical therapy: Part I. Circulation.

[53] Tang Y, Jiang H, Bryan NS (2011). Nitrite and nitrate: cardiovascular risk-benefit and metabolic effect. Curr Opin Lipidol.

[54] Hendgen-Cotta UB, Luedike P, Totzeck M, Kropp M, Schicho A, Stock P (2012). Dietary nitrate supplementation improves revascularization in chronic ischemia. Circulation.

[55] Vilskersts R, Kuka J, Liepinsh E, Cirule H, Gulbe A, Kalvinsh I (2014). Magnesium nitrate attenuates blood pressure rise in SHR rats. Magnes Res.

[56] Stokes KY, Dugas TR, Tang Y, Garg H, Guidry E, Bryan NS (2009). Dietary nitrite prevents hypercholesterolemic microvascular inflammation and reverses endothelial dysfunction. Am J Physiol Heart Circ Physiol.

[57] Rammos C, Hendgen-Cotta UB, Sobierajski J, Bernard A, Kelm M, Rassaf T (2014). Dietary nitrate reverses vascular dysfunction in older adults with moderately increased cardiovascular risk. J Am Coll Cardiol.

[58] Zand J, Lanza F, Garg HK, Bryan NS (2011). All-natural nitrite and nitrate containing dietary supplement promotes nitric oxide production and reduces triglycerides in humans. Nutr Res.

[59] Lidder S, Webb AJ (2013). Vascular effects of dietary nitrate (as found in green leafy vegetables and beetroot) via the nitrate-nitrite-nitric oxide pathway. Br J Clin Pharmacol.

[60] Siervo M, Lara J, Ogbonmwan I, Mathers JC (2013). Inorganic nitrate and beetroot juice supplementation reduces blood pressure in adults: a systematic review and meta-analysis. J Nutr.

[61] Lara J, Ashor AW, Oggioni C, Ahluwalia A, Mathers JC, Siervo M. Effects of inorganic nitrate and beetroot supplementation on endothelial function: a systematic review and meta-analysis. Eur J Nutr. 2015. Epub ahead of print. 10.1007/s00394-015-0872-7 25764393

[62] McVeigh GE, Morgan DR, Allen P, Trimble M, Hamilton P, Dixon LJ (2002). Early vascular abnormalities and de novo nitrate tolerance in diabetes mellitus. Diabetes Obes Metab.

[63] American Diabetes Association (2014). Standards of medical care in diabetes-2014. Diabetes Care.

[64] Fujita K, Wada K, Nozaki Y, Yoneda M, Endo H, Takahashi H (2011). Serum nitric oxide metabolite as a biomarker of visceral fat accumulation: clinical significance of measurement for nitrate/nitrite. Med Sci Monit.

[65] Roberts LD, Ashmore T, Kotwica AO, Murfitt SA, Fernandez BO, Feelisch M (2015). Inorganic nitrate promotes the browning of white adipose tissue through the nitrate-nitrite-nitric oxide pathway. Diabetes.

[66] Larsen FJ, Schiffer TA, Borniquel S, Sahlin K, Ekblom B, Lundberg JO (2011). Dietary inorganic nitrate improves mitochondrial efficiency in humans. Cell Metab.

[67] Ohtake K, Ishiyama Y, Uchida H, Muraki E, Kobayashi J (2007). Dietary nitrite inhibits early glomerular injury in streptozotocin-induced diabetic nephropathy in rats. Nitric Oxide.

[68] Carlstrom M, Persson AE, Larsson E, Hezel M, Scheffer PG, Teerlink T (2011). Dietary nitrate attenuates oxidative stress, prevents cardiac and renal injuries, and reduces blood pressure in salt-induced hypertension. Cardiovasc Res.

[69] Lundberg JO, Larsen FJ, Weitzberg E (2011). Supplementation with nitrate and nitrite salts in exercise: a word of caution. J Appl Physiol (1985).

[70] Greenway FL, Predmore BL, Flanagan DR, Giordano T, Qiu Y, Brandon A (2012). Single-dose pharmacokinetics of different oral sodium nitrite formulations in diabetes patients. Diabetes Technol Ther.

[71] Mohler ER, Hiatt WR, Gornik HL, Kevil CG, Quyyumi A, Haynes WG (2014). Sodium nitrite in patients with peripheral artery disease and diabetes mellitus: safety, walking distance and endothelial function. Vasc Med.

